# Oral syphilis - the great imitator: a series of six cases

**DOI:** 10.1038/s41415-024-7891-9

**Published:** 2024-10-11

**Authors:** Brian Maloney, Claire M. Healy

**Affiliations:** Division of Oral and Maxillofacial Surgery, Oral Medicine, Oral Pathology and Oral Radiology, Dublin Dental University Hospital, School of Dental Science, Trinity College Dublin, Dublin 2, Ireland

## Abstract

Syphilis is an infectious disease which can present with multitudinous mucocutaneous manifestations. Often referred to as the ‘great mimicker', syphilis can present with non-specific symptoms and has a tropism for various organ systems. The oral cavity has been identified as a site commonly affected in the early stages of syphilis infection. Identification of the diverse presentations seen across the different stages of syphilis infection can assist in early diagnosis and treatment for this cohort of patients. Despite accurate diagnostic tools and the susceptibility of the infection to standard antimicrobial therapy, syphilis infections continue to rise worldwide.

We present the clinical features and management of six cases of oral syphilis who presented to our unit. One case presented in 2008, but the other five cases presented between 2016 and 2023, reflecting the increasing incidence of syphilis infection. Five cases presented in the secondary stage of the infection while one presented with a primary infection in the form of a single chancre.

The documented cases demonstrate the non-specific and variable clinical features of oral syphilis and highlight the importance of awareness in the dental profession of these manifestations. Dentists have an important role to play in recognising the disease and arranging appropriate testing for early intervention. This will not only reduce the incidence of the devastating consequences of tertiary infections but will also result in reduced spread of the disease.

## Key points

- The incidence of syphilis has increased worldwide over the last two decades.
- Oral manifestations may be the only or most significant feature of primary and secondary syphilis.
- It is important that the general dental practitioner considers the possibility of this diagnosis in cases of single isolated ulcers (the chancre of primary syphilis) or in widespread oral ulceration or granulomatous disease (secondary syphilis).
- The general dental practitioner plays an important role in the diagnosis and control of syphilis infection as the features of primary and secondary syphilis are self-limiting. A patient who remains undiagnosed is at risk of contributing to further spread of the disease and of developing tertiary syphilis.

## Introduction

Syphilis is a highly contagious disease associated with infection by the anaerobic filamentous spirochete *Treponema pallidum* subspecies *pallidum*.^1^ While vertical transmission can occur *in utero* or peripartum, the infection is most commonly acquired through sexual contact, and, less commonly, through direct vascular inoculation/cutaneous contact with infectious lesions.^2^

Syphilis is a notifiable infectious disease, which has demonstrated a resurgence in the last two decades. While rates of new infection had dropped significantly in the post-penicillin era, cases have steadily increased year-on-year since the onset of the twenty-first century. Despite efforts to reduce rates of new infection, studies have demonstrated the continued high prevalence of syphilis worldwide.^3^^,^^4^

Syphilis is often referred to as the ‘great imitator' for its multitudinous and diverse mucocutaneous manifestations, commonly mimicking other conditions, making it vulnerable to misdiagnosis.^1^ *T. pallidum* has a tropism for the neurological and cardiovascular systems, leading to life-threatening complications if not diagnosed and treated promptly.^5^ Oral manifestations of syphilitic infection have been reported to be an early sign of the disease and can be seen throughout the various stages of the infection.

This case series reports six patients who presented with oral manifestations of syphilis and documents their investigation and management.

## Cases

### Case 1

A patient in their fifties was referred to the oral medicine department by their general dentist in 2008 regarding lower labial sulcus ulceration, present for three months. They denied skin/genital involvement. Medically, they were fit and well. They never smoked and consumed ten units of alcohol per week.

**Fig. 1:**
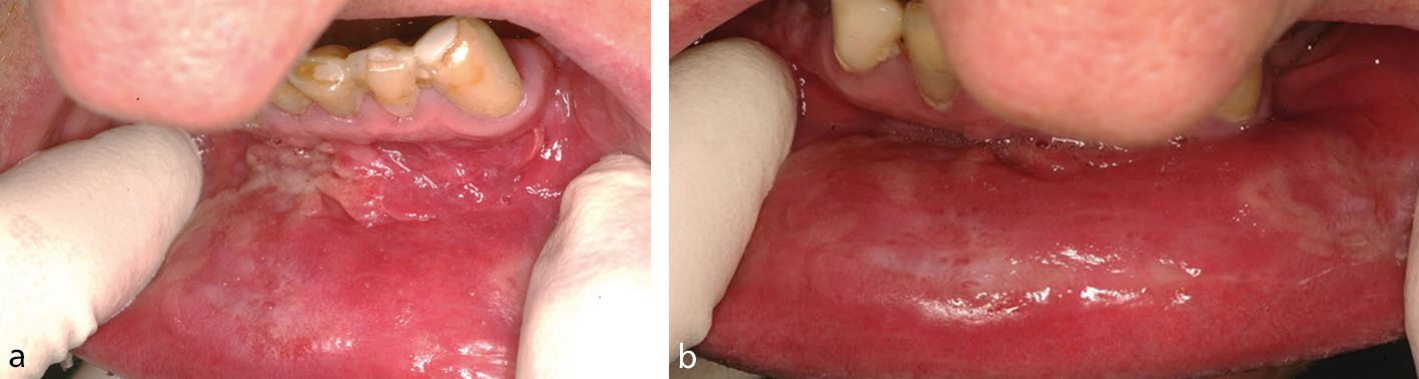
a, b) Case 1

Examination revealed right submandibular lymphadenopathy. There was extensive mucosal tagging and shallow linear ulceration in the lower labial sulcus associated with soft, flat, white mucous patches on the lower labial mucosa (Fig. 1a and b). Differential diagnoses included oral granulomatous disease eg orofacial granulomatosis, syphilis infection, immunobullous disease eg dermatitis herpetiformis, and erosive lichen planus.

Preliminary investigations included full blood count, haematinics, renal and liver profiles, glucose, C-reactive protein (CRP), serum angiotensin-converting enzyme, anti-tissue transglutaminase (tTG) and syphilis serology. Incisional biopsies for histopathology and immunofluorescence were planned but the patient failed to attend the appointment. All bloods were normal apart from syphilis serology, which was positive (rapid plasma reagin [RPR] [1:10], enzyme-linked immunoassay [EIA] [positive], *T. pallidum* particle agglutination assay [TPPA] [1:10240]), indicating a diagnosis of secondary syphilis. The patient was referred to infectious diseases and the case was reported to the Health Protection Surveillance Centre (HPSC) for contact tracing. Screening was carried out for other sexually transmitted diseases. Treatment involved administration of intramuscular benzathine benzylpenicillin.

### Case 2

Case 2 was a patient in their seventies who attended the oral medicine department for a routine review of previously diagnosed linear IgA disease. The patient had rheumatoid arthritis, managed with methotrexate. They were a non-smoker who consumed 14 units of alcohol per week.

On presentation, they reported a severe sore throat of three weeks duration. They had been prescribed a course of phenoxymethylpenicillin but this had no impact and they complained of constant pain and odynophagia.

**Fig. 2:**
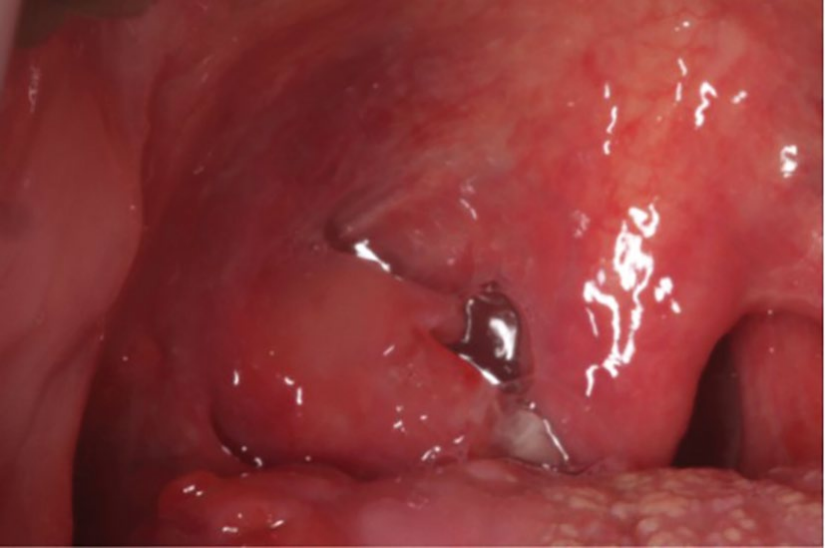
Case 2

On examination, there was no cervical lymphadenopathy. Intra-orally, there was firm ulceration of the right tonsillar region (Fig. 2). The differential diagnosis included oropharyngeal squamous cell carcinoma, non-Hodgkin lymphoma, syphilis infection, or linear IgA disease. Preliminary investigations included bloods (full blood count, liver profile, CRP, albumin), and syphilis serology. An urgent referral to otolaryngology was made for biopsy of the ulcer.

Significant findings included mild lymphopenia of 1.2 (1.5-3.5 × 10^9^ /L) and positive syphilis serology (RPR [1:8], TPPA [1:640], Architect *T. pallidum* chemiluminescence assay [ATPA] [positive]). Given these results, the ulcer was deemed a syphilitic chancre and the biopsy was postponed pending treatment. The patient was referred to infectious diseases and the case was reported to the HPSC. The patient was treated with intramuscular benzathine benzylpenicillin and demonstrated complete resolution of the ulceration.

### Case 3

This patient in their sixties was referred by their general dentist in June 2018 for painful oral ulceration of three months duration. Numerous treatments, including an oral steroid rinse and antifungal agent, had been trialled and had not led to ulcer resolution. Their history was also significant for a rash on the abdomen and back which developed six months prior and lasted for three months. They denied genital involvement. The patient had osteoporosis, hypercholesterolaemia, osteoarthritis and a history of renal calculi. They were taking alendronic acid, ezetimibe and diclofenac acid. They were a never-smoker and non-drinker.

**Fig. 3:**
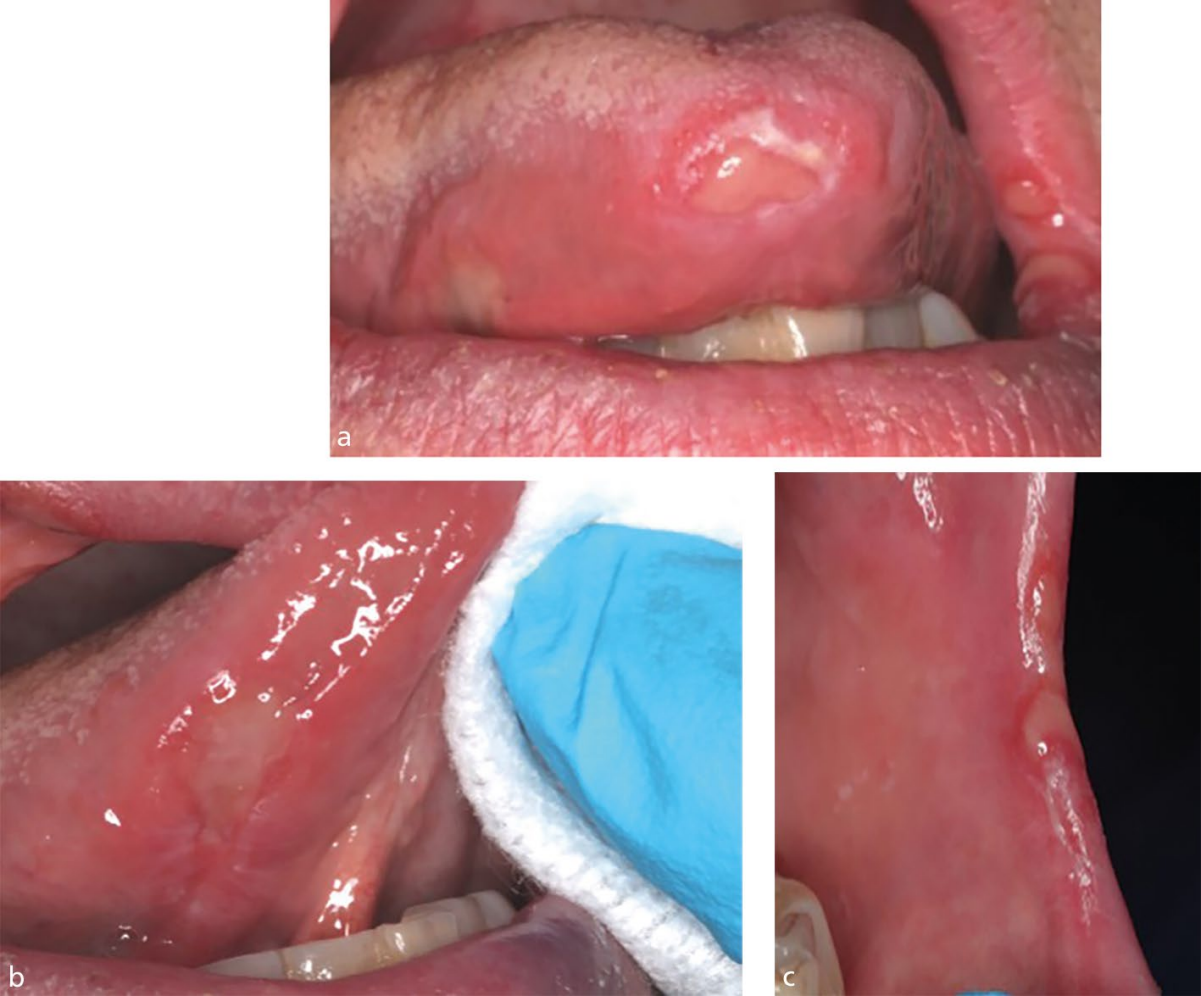
a, b, c) Case 3

On examination, there was no cervical lymphadenopathy. Intra-orally, there were multiple irregular shallow ulcers involving the ventral surface of the tongue, buccal mucosa, floor of the mouth and alveolar ridges (Fig. 3a, b and c). All ulcers were soft and acutely tender. The differential diagnoses included erosive lichen planus, an immunobullous disorder and syphilis.

Viral swabs and a range of blood tests (full blood count, haematinics, haemoglobin A1c [HbA1c], connective tissue disease screen, anti-tTG, renal and liver profiles, CRP) were carried out. Indirect immunofluorescence was also carried out to test for pemphigus vulgaris and bullous pemphigoid. Tissue samples were taken for histopathology and immunofluorescence.

These investigations identified low folate of 2.2 ug/L (normal range: 4.5-20 ug/L). Swabs were negative while histopathology revealed acutely inflamed ulcerated squamous mucosa with an underlying dense plasmacytic inflammatory infiltrate. Direct immunofluorescence demonstrated patchy intracellular staining with IgG, with patchy granular C3 along the dermal-epidermal junction. Immunohistochemistry for *T. pallidum* was positive. Syphilis serology was positive (RPR [1:145], EIA [positive], TPPA [1:842]).

The patient was referred to infectious diseases and it emerged that they were already known to the team as they had been diagnosed with syphilis four months previously. They had failed to disclose this information to the oral medicine team. They had not completed their anti-syphilis regimen at that time. They were again treated with benzylpenicillin but again failed to complete the course, and the serum RPR test did not fall. Therefore, they proceeded to a lumbar puncture, which showed lymphocytes and increased protein in the cerebrospinal fluid (CSF) and CSF-RPR of 1:2, indicating early neurosyphilis, though there were no neurological deficits. The patient was admitted for a two-week course of intravenous benzylpenicillin and had an excellent response, following completion of this regime with no further recurrences of oral or neurosyphilis.

### Case 4

This patient in their seventies was referred to the emergency department by their dentist concerning widespread oral ulceration of six weeks duration. Previous treatments included antibiotics, antifungals and over-the-counter mouthwash. They denied any skin or genital involvement. Medically, the patient had diabetes mellitus, emphysema, hypertension and a penicillin allergy. Their medications included insulin, sitagliptin/metformin, lercanidipine, nebivolol, atorvastatin, clopidogrel and indacaterol/glycopyrronium inhaler. They smoked ten cigarettes per day and rarely consumed alcohol.

**Fig. 4:**
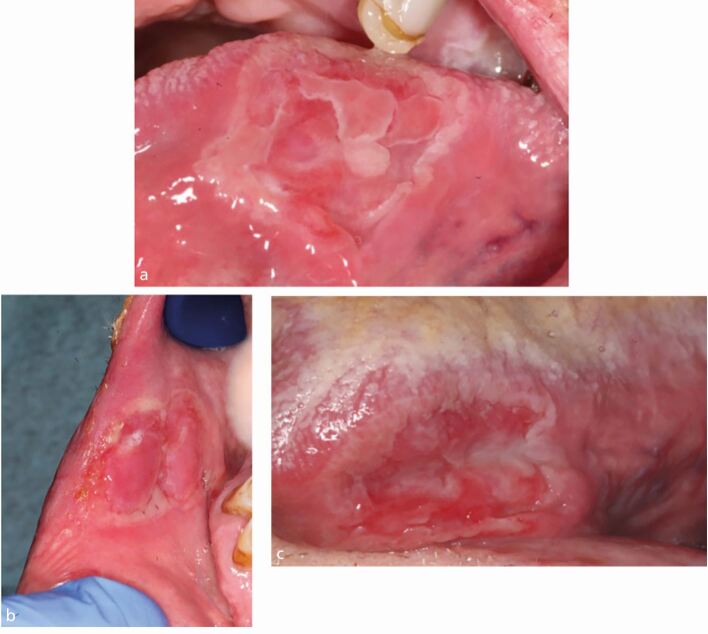
a, b, c) Case 4

Examination revealed bilateral crusting of the angles of the mouth. Intra-orally, there was widespread shallow ulceration involving the buccal and labial mucosa, hard palate and tongue (Fig. 4a, b and c). Differential diagnoses included an immunobullous disorder, erosive lichen planus, recurrent aphthous ulceration and syphilis.

The preliminary workup consisted of a full blood count and haematinics (to rule out haematinic deficiency-related oral ulceration), connective tissue disease screen, renal and liver profiles, HbA1c and syphilis serology. Serology for syphilis was positive (RPR [1:256], TPPA [1 ≥ 2,560], EIA [positive], ATPA [positive]), confirming the diagnosis of oral syphilis. Other significant findings included mild lymphopenia of 1.3 (1.5-3.5 × 10^9^/L), an elevated HbA1c of 53 (20-42 mmol/mol), elevated gamma-glutamyl transferase of 84 (10-71 IU/L) and CRP of 22.4 (0-5 mg/L). The patient was referred to infectious diseases and the case was notified to the HPSC. Given their penicillin allergy, they were treated with doxycycline which resulted in the resolution of the oral ulceration.

### Case 5

This patient in their sixties was referred to the oral medicine department by their general dentist regarding asymptomatic ulceration involving the right and left lateral borders of the tongue. The ulceration was an incidental finding by the patient's dentist who initially believed the cause to be traumatic. An occlusal splint was made for the patient. However, on review one month later, the ulcer on the left lateral border of the tongue had resolved but the ulcer on the right persisted and prompted referral. The patient denied any skin or genital symptoms. The patient had HIV (human immunodeficiency virus) disease, with an undetectable viral load and previous infection with hepatitis B. Their medications included dolutegravir/abacavir/lamivudine and aspirin. They were a non-smoker who consumed 28 units of alcohol per week.

**Fig. 5:**
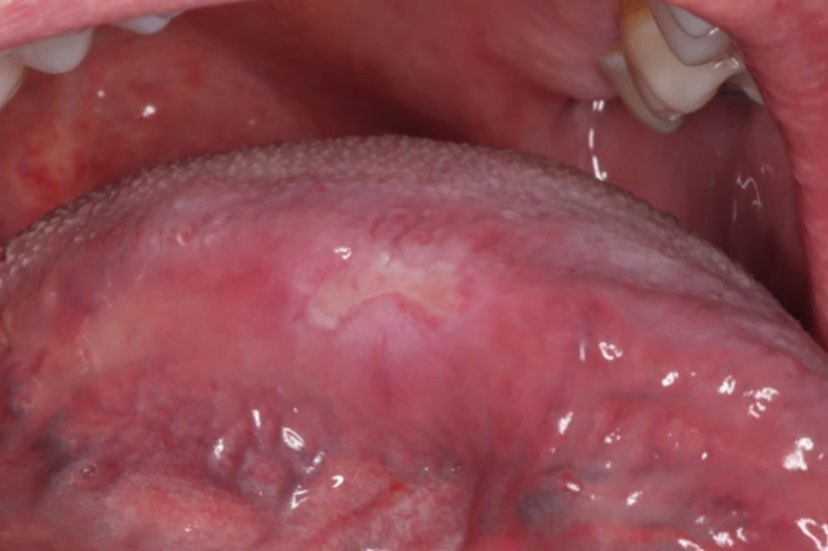
Case 5

There was no cervical lymphadenopathy. There was a single, soft, tender ulcer of the right lateral border of the tongue measuring 10 x 4 mm with no obvious cause (Fig. 5). The differential diagnosis included a traumatic ulcer and an early squamous cell carcinoma.

An incisional biopsy of the ulcer was taken and revealed ulcerated inflamed squamous epithelium with a florid plasma cell-predominant inflammatory infiltrate in the underlying connective tissue stroma. Immunohistochemical antibody was positive for syphilis. They proceeded to syphilis serology, which was positive (RPR [1:64], TPPA [1:648], EIA [positive], ATPA [positive]), thus confirming the diagnosis of secondary syphilis. The patient's infectious diseases team was informed and the HPSC was notified. They had a course of intramuscular benzylpenicillin and had an excellent response to treatment.

### Case 6

Case 6 was a patient in their fifties who presented to the emergency department with pain in the tongue and lips, which began one month previously. They denied any skin or genital involvement. They were fit and well and on no medication. They smoked 15 cigarettes per day for 40 years and did not consume any alcohol.

**Fig. 6:**
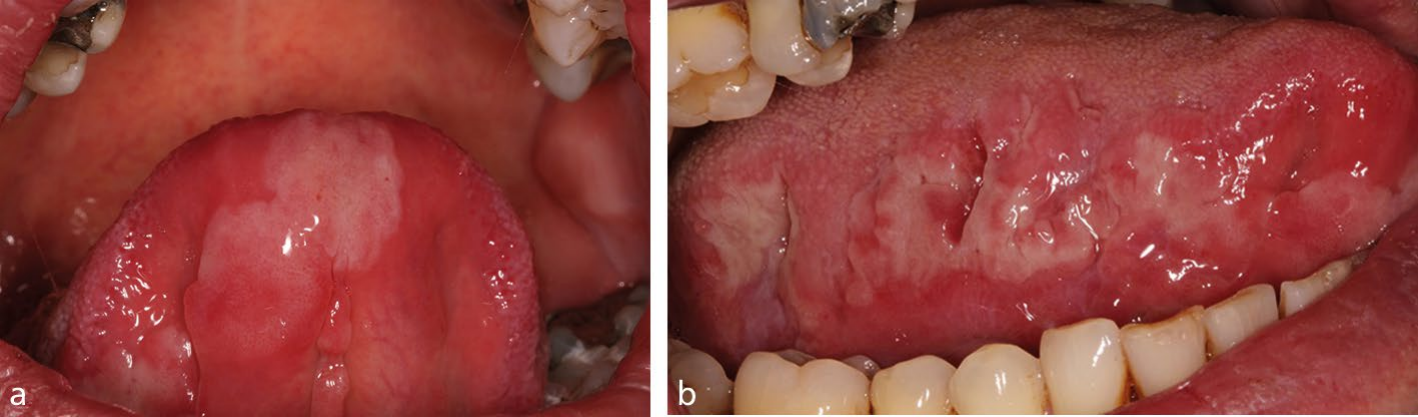
a, b) Case 6

Examination revealed tender left submandibular lymphadenopathy. Intra-orally, there was extensive ulceration of the right and left lateral borders, dorsum and ventral surface of the tongue, labial mucosa, buccal mucosa, and palate, all of which were soft and tender to palpate (Fig. 6a and b). The differential diagnosis included herpetic ulceration, erythema multiforme, immunobullous disease, syphilis infection and erosive lichen planus.

Preliminary investigations included a full blood count, haematinics, renal and liver profiles, CRP, HbA1c, connective tissue disease screen, immunoglobulin levels, syphilis serology and viral swabs of the ulceration. Syphilis serology was positive (RPR [1:85], TPPA [1 > 2,560], EIA [positive], ATPA [positive]), thus confirming the diagnosis of oral syphilis. Serum IgG was elevated at 18.68 (6.26-14.96 g/L). The patient was referred to infectious diseases and was successfully treated with intramuscular penicillin. The HPSC was notified of the case.

A summary of the workup for all cases and their management can be seen in Table 1.

**Table 1:** Summarising table of Cases 1 to 6

Case	Age range	Stage	Clinical features	Differential diagnosis	Treatment	Follow-up
1	50-60	Secondary	Regional lymphadenopathy, mucosal tagging, extensive shallow oral ulceration	Granulomatous disease (orofacial granulomatosis, oral Crohn's disease, sarcoidosis) Syphilis Immunobullous disease Erosive lichen planus	Referral to infectious diseases (ID) Case reported to the HPSC for contact tracing Treatment with intramuscular (IM) benzathine benzylpenicillin	No recurrence of oral syphilis on review
2	70-80	Primary	Firm ulcer of right tonsillar region	Oropharyngeal squamous cell carcinoma Non-Hodgkin lymphoma Syphilis Linear IgA disease	Referral to ID Case reported to the HPSC for contact tracing Treatment with IM benzathine benzylpenicillin	Complete resolution of oral syphilis on review
3	60-70	Secondary	Multiple irregular shallow ulcers	Erosive lichen planus Syphilis Immunobullous disease	Referral to ID Case reported to the HPSC for contact tracing Treatment with IM benzathine benzylpenicillin	Failed to complete treatment with IM penicillin; subsequently developed early neurosyphilis (detected with lumbar puncture) Admitted to hospital for treatment with IV benzylpenicillin Complete resolution of oral syphilis and neurosyphilis
4	70-80	Secondary	Widespread shallow ulceration	Immunobullous disease Erosive lichen planus Recurrent aphthous ulceration Syphilis	Referral to ID Case reported to the HPSC for contact tracing Treatment with doxycycline (penicillin allergy)	Complete resolution of oral syphilis on review
5	60-70	Secondary	Single, soft, ulcer of the right lateral border of the tongue	Traumatic ulcer Early oral squamous cell carcinoma	Referral to ID Case reported to the HPSC for contact tracing Treatment with IM benzylpenicillin	Complete resolution of oral syphilis on review
6	50-60	Secondary	Submandibular lymphadenopathy. Extensive ulceration	Viral ulceration Erythema multiforme Syphilis Erosive lichen planus Immunobullous disease	Referral to ID Case reported to the HSPC for contact tracing Treatment with IM benzylpenicillin	Complete resolution of oral syphilis on review

## Discussion

Syphilis is a complex, ancient disease which has experienced a resurgence in the last two decades.^6^ The disease is a good candidate for eradication, given its host is strictly human, alongside the availability of diagnostic tests and effective treatment regimes. However, ongoing efforts to eliminate the disease have failed and syphilis remains a public health problem in both developed and developing countries worldwide.^7^

In 2001, the incidence of syphilis was at its lowest rate since the beginning of surveillance of the disease.^1^ Recent years, however, have seen a significant increase in cases. Case-based notifications of early syphilis infection in Ireland have increased each year since 2013.^8^ As of 2022, there were 892 notifications, of which 94% were male, a 13.9% increase from the previous year. Similarly in the UK, rates of new syphilis diagnoses in 2022 were the highest since disease surveillance began in 1948; a 15% increase from 2021.^7^ Gay, bisexual and other men who have sex with men remain the groups most affected by new exposures to syphilis. This increase reflects trends worldwide.

Alongside this increase in cases of infection, oral manifestations are also occurring at a high frequency.^9^ Given that oral manifestations may be the initial presentation of the disease, dentists play an important role in the early recognition, diagnosis and treatment of infection. Delayed or missed diagnosis leaves patients at risk of tertiary disease consequences, which can be life-threatening. It also results in further disease transmission via patient sexual contact.^10^

Considerable challenges in the management of syphilis include its prolonged clinical course, often asymptomatic presence and the disease's protean manifestations, thereby contributing to the progression and spread of the disease.^1^In addition, infection does not confer immunity, resulting in re-infection upon re-exposure following treatment.

### Clinical characteristics

Syphilis demonstrates four overlapping characteristic stages based on the time elapsed since exposure. Each stage has distinct clinical features and degrees of infectivity. Clinical manifestations result from local inflammatory responses to replicating spirochetes and often imitate those of other diseases.^11^Table 2 summarises the clinical stages of syphilis infection.

**Table 2:** Stages of syphilis infection

Primary	Solitary, non-exudative ulcer with base infiltration and hardened high margins, referred to as a ‘chancre'. Most commonly involve the gingiva, tongue and lips, and develop three weeks after inoculation Non-tender regional lymphadenopathy
Secondary	Raised erosions of the oral mucosa with an overlying pseudomembranous slough known as ‘mucous patches' and distinctive ‘snail track' ulcers affecting multiple oral sites Granulomatous swellings are less commonly described Maculopapular rash of varying extensions may occur on the skin due to spirochaetal haematological dissemination
Latent	Usually asymptomatic, with no mucocutaneous manifestations
Tertiary	Gumma: painless, granulomatous inflammation involving the palate and/or tongue Atrophic luetic glossitis Syphilitic leukoplakia Cardiac or neurological conditions, severe skin or visceral lesions or bony involvement

Primary syphilis is associated with a solitary, non-exudative ulcer with base infiltration and hardened high margins, referred to as a ‘chancre', as seen in Case 2. Chancres most commonly involve the gingiva, tongue and lips, and develop three weeks after inoculation.^1^ Primary infection is also associated with non-tender regional lymphadenopathy which may be mistaken for metastases.^12^

In secondary disease, oral manifestations are most common, affecting over 20% of cases and are also the most diverse.^13^ This stage develops 6-8 weeks after the resolution of the chancre ulcer.^14^ Five of our cases presented with manifestations of secondary syphilis. This stage is marked by a spirochetaemia with wide dissemination. The dissemination of spirochetes can result in a maculopapular rash of varying extension, as seen in Case 3. This stage resolves spontaneously and the patient enters another latency period.^15^ Secondary syphilis may demonstrate raised erosions of the oral mucosa with an overlying pseudomembranous slough known as ‘mucous patches', as seen in Case 1, and distinctive ‘snail track' ulcers, as seen in Case 4, affecting multiple oral sites. Granulomatous swellings, as seen in Case 1, have been less commonly described.

The latent phase of the disease is considered inactive but with serologic proof of infection.^2^It is often asymptomatic, with no mucocutaneous manifestations.

If the disease is not diagnosed and treated appropriately, syphilis can progress to the tertiary phase, which is a multi-organ stage of infection. Historical literature suggests that 15-40% of untreated individuals will develop tertiary syphilis, which can manifest as destructive cardiac or neurological conditions, severe skin or visceral lesions, or bony involvement.^16^ Oral features of tertiary syphilis have been characterised by gumma (painless, granulomatous inflammation involving the palate and/or tongue), atrophic luetic glossitis, and more rarely, by syphilitic leukoplakia.^17^ However, it is unclear whether this entity is truly associated with syphilis infection, or whether it is in fact secondary to a smoking habit.^17^ This stage of disease has become a rarity as a result of effective antibiotic treatment.

### Differential diagnosis

Clinical correlation is important in cases of suspected syphilis as the disease may mimic a range of other unrelated conditions. When present in its primary form as a chancre, syphilis can resemble a traumatic ulcer, carcinoma, major aphthous ulcer, tuberculosis, drug-induced ulceration or deep fungal infection. Secondary syphilis manifestations are more diverse and may resemble a range of both infectious and non-infectious conditions, including herpetic infection, immunobullous disease, ulcerative lichen planus and drug-induced ulceration. Gumma in the tertiary stage can mimic destructive pathologies, such as oral lymphoma or granulomatosis with polyangiitis. Tertiary syphilis may also present as a white patch, termed ‘syphilitic leukoplakia'.

### Diagnosis

Detection of *T. pallidum* can be challenging as the bacterium cannot be cultured *in vitro*. Laboratory-based diagnosis of syphilis infection is achieved by direct visualisation of the bacterium in clinical specimens or through assessment of the reactivity of patient serum in serological tests.^18^ Biopsy of affected tissue may, however, be indicated to rule out other pathology, as well as allow the identification of *T. pallidum* by immunohistochemistry. The histologic features of syphilis-infected tissues are often non-specific^6^ but commonly demonstrate characteristic infiltration with plasma cells in the lamina propria and deeper stroma. This dense infiltrate surrounds nerves and blood vessels. There can be ulceration as well as fibropurulent exudate. In cases of tertiary syphilis, chronic granulomatous inflammation is present.

Visualisation of *T. pallidum* has been achieved, traditionally, through dark field microscopy with scrapings of exudate from active lesions or, in modern times, through immunohistochemistry biopsy samples. The latter is more commonly employed and may be beneficial for diagnosis in the early stages of infection where antibodies may be undetectable via serology. Anti-*T. pallidum* immunohistochemistry demonstrates the characteristic appearance of corkscrew-shaped spirochetes infiltrating the epithelium, alongside a perivascular pattern in the deeper stroma.

There are two broad categories of blood tests for the detection of *T. pallidum*: treponemal and non-treponemal (NT) assays. These measure different antibody reactivities.^18^ NT tests, also known as flocculation assays, determine the reactivity of patient serum to cardiolipin-cholesterol-lectin antigen released from damaged host cells. Common NT tests include venereal disease research laboratory (VDRL) test, RPR and toluidine red unheated serum test.^19^ These tests are associated with false positive responses so they must be followed-up by confirmatory treponemal tests. Treponemal tests employ the use of *T. pallidum* antigens and detect immunoglobulin to treponemal components.^18^ They include the fluorescent treponemal antibody absorption assay (FTA-ABS), rapid point-of-care immunochromatographic strip assay, TPPA, EIA and chemiluminescence immunoassay. Treponemal tests are considered more sensitive than NT for all stages of infection but are not useful for monitoring infection as they cannot differentiate between past or treated infection and active infection.^19^ These tests are summarised in Table 3.

**Table 3:** Syphilis serological tests

**Non-treponemal tests: detect antibodies directed at lipoidal antigens, as well as host cells damaged from syphilis infection**	
Venereal disease research laboratory test (VDRL)	Detects level of anti-lipid immunoglobulin to lipoidal material (cardiolipin) released from damaged host cells or *T. pallidum*
Rapid plasmin reagin (RPR)	Flocculation assay, semi-quantitative. Detects non-treponemal immunoglobulins to lipoidal material (cardiolipin) released from damaged host cells or *T. pallidum*
**Treponemal tests: detect reactivity of anti-treponemal antibodies against antigens of T. pallidum**	
*T. pallidum* particle agglutination assay (TPPA)/*T. pallidum* haemagglutination assay (TPHA)	Qualitative indirect gelatine particle or red cell agglutination tests. Detect immunoglobulins against *T. pallidum* antigens
Fluorescent treponemal antibody absorbed FTA-ABS test	Indirect immunofluorescent assay. Antigens of *T. pallidum* exposed to patient serum (following mixture with sorbent). Anti-human immunoglobulins combine with antibodies in the patient's serum if present. Visualised by fluorescence microscopy
Enzyme Immunoassay (EIA)	Enzyme-linked immunoassay for detection of anti-treponemal IgG/IgM
Chemiluminescence immunoassay	Light-based immunoassay that detects anti-*T. pallidum* antibodies (IgG/IgM), e.g. Architect *T.pallidum* assay

There is no agreed protocol for serological investigation of syphilis. However, the traditional algorithm involves a combination of non-treponemal and treponemal tests, followed-up with a treponemal test of similar sensitivity and specificity in positive cases.^20^ Where positive cases are detected, repeat testing should be done and results sent to a public health laboratory for collection and reference testing.

### Treatment

A breakthrough in the management of syphilis occurred with the first report of successful treatment of syphilis with penicillin.^21^ Fortunately, *T. pallidum* has remained sensitive to penicillin preparations. As such, long-acting penicillin preparations via intramuscular administration are the first-line drug employed for all stages of syphilis in non-allergic patients.^22^ The dosage and duration of treatment are dictated by the stage and severity of the infection.^20^ Response to treatment is regularly monitored via serological re-evaluation of non-treponemal titre levels. Alternatives to penicillin in cases of allergy include macrolides, tetracyclines and ceftriaxone.

The incidence of HIV/syphilis co-infection has increased in recent years, ranging between 8-25% of total cases.^23^ Untreated syphilis has been reported to have a synergistic effect with HIV infection, resulting in aggressive and atypical syphilis sequelae, including syphilitic uveitis and neurosyphilis.^24^ Patients with co-infection with HIV should be treated in the same manner as those without HIV.

### Congenital syphilis manifestations

Congenital syphilis is classified as early and late based on the onset of clinical symptoms and can demonstrate diverse clinical manifestations affecting the skeletal, neurological, ocular, reticuloendothelial and cutaneous systems. Early features include hepatosplenomegaly, jaundice, periostitis, rhinitis, lymphadenopathy and rash.^25^ In cases of late congenital syphilis, sensorineural loss of hearing and dental anomalies, such as notched incisors (‘Hutchinson's incisors'), dome-shaped and noduled molars (‘mulberry molars'), can be observed.^17^ It is also important to note that congenital syphilis is a marker of heterosexual spread of syphilis infection.

### Complications of untreated syphilis

Untreated syphilis can have life-threatening complications for patients.^26^^,^^27^ Mortality associated with untreated infection is also high, at a rate of 8-14% over a 30-year period.^26^ A total of 75% of cases followed for 15 years without treatment developed the tertiary stage of disease, with 50% of cases suffering cardiovascular complications.^27^ Syphilis can result in inflammation-mediated damage, commonly resulting in aneurysms and coronary artery disease. Invasion of the central nervous system with *T. pallidum* can occur at any stage of infection, and while uncommon, can cause meningovascular syphilis, cranial nerve palsies and paresis.^28^ Nerve palsies and neuropathies of the trigeminal and facial nerve, either unilaterally or bilaterally, are rare, but have been described in the literature.^29^

## Conclusion

The prevalence of syphilis is increasing worldwide, despite continuous public health efforts to eradicate the disease. The variability of the presentation of the disease creates a challenge for clinicians in reaching a prompt diagnosis. Our case series serves to highlight the non-specific nature of syphilis-related oral manifestations. All patients were over 50 years of age, indicating the importance of considering sexually transmitted disease in the differential diagnosis of oral and systemic disease in older patients. Awareness of the range of oral features seen in patients with syphilis infection may facilitate dentists to recognise the disease and arrange appropriate testing for early intervention. Enhanced screening, contact tracing, improved surveillance and education are also fundamental to limiting the spread of this age-old disease.
